# Stabilizing Non‐Fullerene Organic Photodiodes through Interface Engineering Enabled by a Tin Ion‐Chelated Polymer

**DOI:** 10.1002/advs.202302976

**Published:** 2023-08-04

**Authors:** Jianhua Xiao, Yang Wang, Liu Yuan, Yin Long, Zhi Jiang, Qingxia Liu, Deen Gu, Weizhi Li, Huiling Tai, Yadong Jiang

**Affiliations:** ^1^ State Key Laboratory of Electronic Thin Films and Integrated Devices School of Optoelectronic Science and Engineering University of Electronic Science and Technology of China Chengdu 610054 China; ^2^ Innovative Center for Flexible Devices (iFLEX) School of Materials Science and Engineering Nanyang Technological University 50 Nanyang Avenue Singapore 639798 Singapore

**Keywords:** cathode interfacial layers, interface engineering, non‐fullerene acceptors, organic photodiodes, stability

## Abstract

The recent emergence of non‐fullerene acceptors (NFAs) has energized the field of organic photodiodes (OPDs) and made major breakthroughs in their critical photoelectric characteristics. Yet, stabilizing inverted NF‐OPDs remains challenging because of the intrinsic degradation induced by improper interfaces. Herein, a tin ion‐chelated polyethyleneimine ethoxylated (denoted as PEIE‐Sn) is proposed as a generic cathode interfacial layer (CIL) of NF‐OPDs. The chelation between tin ions and nitrogen/oxygen atoms in PEIE‐Sn contributes to the interface compatibility with efficient NFAs. The PEIE‐Sn can effectively endow the devices with optimized cascade alignment and reduced interface defects. Consequently, the PEIE‐Sn‐OPD exhibits properties of anti‐environmental interference, suppressed dark current, and accelerated interfacial electron extraction and transmission. As a result, the unencapsulated PEIE‐Sn‐OPD delivers high specific detection and fast response speed and shows only slight attenuation in photoelectric performance after exposure to air, light, and heat. Its superior performance outperforms the incumbent typical counterparts (ZnO, SnO_2_, and PEIE as the CILs) from metrics of both stability and photoelectric characteristics. This finding suggests a promising strategy for stabilizing NF‐OPDs by designing appropriate interface layers.

## Graphical abstract

A tin ion‐chelated polyethyleneimine ethoxylated (PEIE‐Sn) is prepared as a generic cathode interfacial layer of non‐fullerene organic photodiodes (NF‐OPDs). The PEIE‐Sn can endow the NF‐OPDs with improved interface compatibility, optimized cascade alignment, and reduced interface defects. Consequently, the PEIE‐Sn‐OPD shows performance that outperforms the incumbent typical counterparts (based on other CILs) from metrics of both stability and photoelectric characteristics.

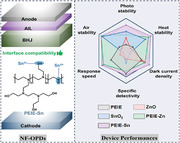

## 1 Introduction

Organic photodetectors are emerging as a promising alternative to the traditional inorganic counterparts in optical detection technology, thanks to their remarkable advantages,^[^ ^1^, ^2^, ^3^, ^4^ ^]^ and thereby have potential applications in health monitoring, biomedical imaging, artificial vision, and so on.^[^ ^5^, ^6^, ^7^, ^8^, ^9^ ^]^ In general, the state‐of‐the‐art organic photodetector is diode‐type (that is organic photodiode, OPD), which consists of a bulk‐heterojunction (BHJ) active layer that is in the form of phase‐separated blends of electron donor and electron acceptor.^[^ ^10^ ^]^ Among them, the recent emergence of non‐fullerene acceptors (NFAs) has achieved breakthroughs in specific detection (D*) and wide spectrum due to their high absorption coefficient, easy‐to‐customize optical complementarity, and energy compatibility.^[^ ^11^, ^12^ ^]^ However, their operational stability, as an important factor in the commercialization of NF‐OPDs, lags far behind due to their immature development. Fortunately, to some extent, their progress can be inspired by organic photovoltaic (OPV) research. Several factors such as air, light, and heat affect the stability of the devices in two ways: extrinsic and intrinsic degradation,^[^ ^13^ ^]^ where external degradation caused by water and oxygen in the air as traps or reactions with functional layers can be mitigated by high‐quality encapsulation.^[^ ^14^ ^]^ Intrinsic degradation is primarily caused by unstable functional materials, poor film morphology, and poor compatibility between functional layers.^[^ ^15^ ^]^

To refrain from the intrinsic degradations of organic optoelectronic devices, a notable amount of effort has been devoted to the perspectives of optimizing active layers and interfacial engineering. Among them, the strategy for optimizing the active layer comes from the perspectives of the active layer materials and thin films. On the one hand, stable organic materials are obtained through material design, such as optimizing the geometric structure of receptor materials,^[^ ^16^ ^]^ preparing multicomponent photoactive layer based on one‐pot polymerization, and studying oligomer receptors.^[^ ^17^, ^18^ ^]^ On the other hand, stable films are generally obtained by controlling the morphology through the use of multiphase structures or additives.^[^ ^19^, ^20^ ^]^ In addition, poor interface layers may introduce impurities into other functional layers, resulting in high carrier recombination and low device stability.^[^ ^21^, ^22^ ^]^ Therefore, preparing reliable interfaces, especially cathode interfacial layers (CILs), play a critical role in extending the device's lifetime.^[^ ^23^, ^24^, ^25^ ^]^ Among all CILs, ZnO in metal oxides (ZnO, SnO_2_, etc.) has attracted much attention due to its wide band gap, high electron mobility, and environment‐friendly characteristics.^[^ ^26^, ^27^ ^]^ However, recent studies have confirmed that light radiation can weaken its charge transfer selectivity and lead to the degradation of NFAs.^[^ ^23^, ^25^, ^28^ ^]^ Moreover, its inherent property of brittleness and high‐temperature annealing process limits its application in flexible devices. Organic CILs, such as polyethyleneimine ethoxylated (PEIE) and polyethyleneimine (PEI) electrolytes, have been widely used in inverted optoelectronic devices, as the aliphatic amine groups in PEIE can significantly reduce the work function (WF) of the cathode to achieve energy level alignment.^[^ ^29^, ^30^, ^31^, ^32^ ^]^ However, the hydrophilic tail of PEIE deteriorates the device stability, and it has also been proven to reduce device performance by disrupting the chemical structure and intra‐molecular charge transfer of NFAs.^[^ ^27^, ^31^ ^]^ Furthermore, the limitation of insulation characteristics restricts their thickness to less than 10 nm in devices, otherwise unfavorable for the transport of photo‐generated carriers.^[^ ^33^ ^]^ To address the aforementioned issues, Yang et al. reported a zinc ion chelated PEI (PEI‐Zn) CIL that exhibits mechanical flexibility, chemical compatibility with NFAs, and film thickness tolerance and enhances the efficiency of OPVs. Nevertheless, considering the preparation of the PEI‐Zn layer involves a procedure of annealing in air, the excess Zn^2+^ ions are speculated to change into ZnO,^[^ ^27^ ^]^ which may reduce the stability of the NFA‐based devices. In summary, CIL plays a critical role in determining the performance of OPDs, but interface‐related device stability has not been thoroughly studied. Therefore, a well‐designed CIL that is compatible with the photoactive layer and beneficial for both the efficiency and stability of OPDs is essential.

**Figure 1:**
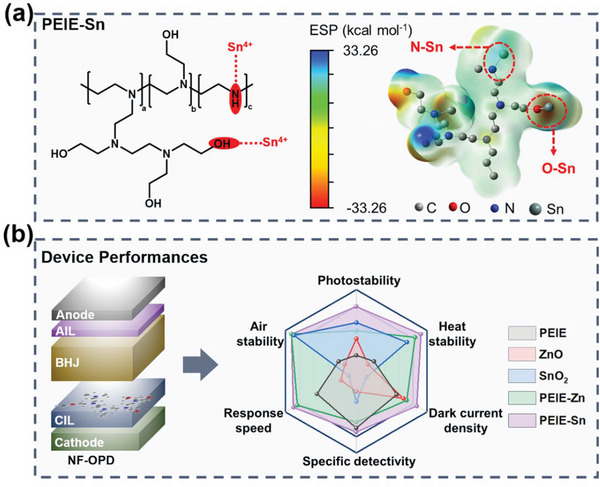
Summary of PEIE‐Sn‐based OPDs. a) Chemical structure (left) and electrostatic potential distribution (right) of PEIE‐Sn. b) Architecture and performance comparison diagram of inverted OPDs with different CILs.

In this work, we report an organometallic CIL of PEIE‐Sn (tin ion‐chelated PEIE) and demonstrate its ability to stabilize inverted NF‐OPDs by interface engineering. The chelation between tin ions (Sn^4+^) and nitrogen/oxygen atoms in PEIE‐Sn contributes to the interface compatibility with efficient NFAs (**Figure** **1**a). Compared with OPDs based on typical CILs layers (PEIE, ZnO, SnO_2_, and PEIE‐Zn), the PS‐OPD (OPD with PEIE‐Sn as CIL) shows merits of anti‐environmental interference, suppressed dark current, accelerated interfacial electron extraction and transmission, thus exhibiting superior photoelectric performance and stability (Figure 1b). The universality of PEIE‐Sn is also demonstrated in several NF‐OPDs. This study provides a promising CIL for stabilizing NF‐OPDs and indicates a general strategy of interface engineering for investigating organic optoelectronic devices.

## 2 Results

### 2.1 Structural and Morphology Characterizations of PEIE‐Sn

**Figure 2:**
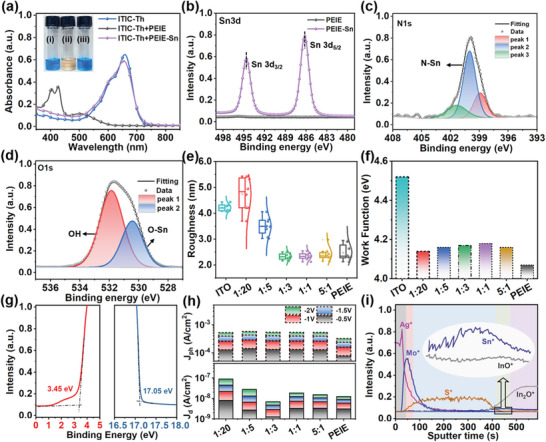
Characterization and chelation ratio optimization of PEIE‐Sn. a) Absorption spectra and optical photos (inset) of i) ITIC‐Th, ii) ITIC‐Th/PEIE, and iii) ITIC‐Th/PEIE‐Sn solutions. XPS spectra of b) Sn 3d of PEIE‐Sn and PEIE, c) N 1s, and d) O 1s of PEIE‐Sn, respectively. e) Roughness and f) work function comparison diagrams of PEIE‐Sn with different M_N_/M_Sn_. g) UPS profiles of (left) valence region and (right) secondary electron cut‐off region (PEIE‐Sn with M_N_/M_Sn_ of 1:3). h) Distribution of photocurrent density (*J_ph_*) and dark current density (*J_d_*) of PEIE and PS‐OPDs. i) TOF–SIMS spectrometry and the inset show an enlarged view of the curve at the interface on both sides of the PEIE‐Sn layer.

Inverted OPDs with energy level alignment were fabricated and tested under reverse bias (Figure S1, Supporting Information). All the devices comprise a cathode of indium tin oxide (ITO), BHJ of PBDB‐T:ITIC‐Th (donor:acceptor) blend (Figure S2, Supporting Information), anode interface layer of MoO_3_, and anode of silver film. As to the CIL, PEIE‐Sn was obtained by introducing Sn^4+^ into PEIE. The chemical compatibility of PEIE‐Sn with NFAs was investigated while using PEIE as the experimental control. When the ITIC‐Th solution was added to PEIE, the color changed from blue to brown and the distinct absorption band (530–730 nm) disappeared, suggesting a chemical reaction occurs between the two components (**Figure** **2**a). This phenomenon is caused by the amine of PEIE reacting as a nucleophile with the C=C linkage moiety in ITIC‐Th through the Michael addition reaction.^[^ ^31^ ^]^ However, the color and absorption spectra of the NFAs solution show barely any variation after being blended with PEIE‐Sn, indicating superior chemical compatibility between them. Other typical NFAs (Y6 and IT‐4F) were also investigated and similar results were obtained (Figure S3, Supporting Information). This is speculated that the introduced Sn^4+^ can chelate with nitrogen atoms and hydroxyl functional groups, thereby suppressing the chemical activity of PEIE.^[^ ^34^ ^]^

To ascertain our speculation, the electrostatic potential (ESP) analysis related to PEIE and PEIE‐Sn, based on density functional theory (DFT), was performed. Figure S4, Supporting Information exhibits high ESP of N (−28.64 kcal mol^−1^) and O (−27.77 kcal mol^−1^) in PEIE, driving the formation of N–Sn and O–Sn bonds.^[^ ^35^ ^]^ After chelation, the ESP of N and O (in N‐Sn and O‐Sn) reduced to 3.14 and −14.43 kcal mol^−1^, respectively (Figure 1a). This lone pair electron coordination effectively suppresses the chemical reaction between PEIE and NFAs.

To quantitatively optimize the chelation ratio between PEIE and Sn^4+^, the content of C, H, N, and O elements in PEIE was determined by organic element analysis (Table S1, Supporting Information), and the PEIE‐Sn mentioned below is expressed by the molar ratio of N and Sn (M_N_/M_Sn_, ranging from 1:20 to 5:1). Based on this, the N‐Sn and O‐Sn bonds in PEIE‐Sn were further confirmed by X‐ray photoelectron spectroscopy (XPS) analysis. Compared with PEIE, the binding energy corresponding to the Sn 3d, N 1s, and O 1s core levels of PEIE‐Sn shifts to higher values (Figure S5, Supporting Information). Specifically, the Sn 3d (M_N_/M_Sn_ of 1:3) shows two main peaks at 486.6 and 495.0 eV, corresponding to Sn 3d_5/2_ and Sn 3d_3/2_ (Figure 2b), respectively.^[^ ^36^ ^]^ To precisely analyze the difference in chemical bonds of the two films, the N 1s and O 1s peaks were deconvolved into multiple spectra. The N 1s of PEIE features centered at 398.25 and 399.3 eV (peaks 1 and 2) are associated with neutral amine nitrogen at different chemical environments, whereas the feature at 400.6 eV (peak 3) is associated with nitrogen in protonated amine groups (Figure S6a, Supporting Information).^[^ ^31^, ^37^, ^38^ ^]^ The introduction of Sn^4+^ increases the area proportion of peak 2 from 11.96% to 47.47% with marginal shifts, which also weakens peak 3 evidently, indicating that electron transfers from N of PEIE to Sn^4+^ for the coordination (Figure 2c). As such, this electron transfer weakens the electronegativity of amine in PEIE, accordingly inhibiting its chemical reactivity. The O 1s peak (531.3 eV) of PEIE is dominated by the hydroxide species, which usually act as shallow trap sites (Figure S6b, Supporting Information). Nevertheless, as displayed in Figure 2d, the hydroxyl peak strength of PEIE‐Sn is significantly reduced. Additionally, a peak at 530.4 eV originating from the oxygen atom chelated with Sn (Sn‐O‐Sn) appears, which benefits electron transportation.^[^ ^39^ ^]^

Then, we investigated the film quality of PEIE‐Sn by changing the M_N_/M_Sn_. Figure S7, Supporting Information shows that the film thickness, ≈16 nm for the 1:3 sample, decreases with the increase of M_N_/M_Sn_. All the films are found to be visible‐range transparent (surpassing 95%), ensuring that the incident light into the absorber layer with barely less (Figure S8, Supporting Information). As M_N_/M_Sn_ increases, the film root‐mean‐square roughness value decreases gradually at the first stage and then increases slightly (Figure 2e and Figure S9, Supporting Information), and that of the 1:3 sample is relatively small. This is speculated that the number of oxides (such as SnO_2_) contained in PEIE‐Sn gradually decreases at the first stage, conducive to improving the film's flatness until the film is too thin to decorate the rough ITO surface (> 4 nm). Moreover, the scanning electron microscope (SEM) images of 1:3 and 1:20 films also show that the surface of the 1:3 sample is smoother (Figure S10, Supporting Information), which helps in a seamless transition from the CILs to active layers without defects, allowing for better charge extraction.^[^ ^40^, ^41^ ^]^ High‐resolution transmission electron microscopy (HR‐TEM) results suggested that excessive Sn ions in PEIE‐Sn existed in the form of SnO_2_ (Figure S11, Supporting Information).

The WF of PEIE‐Sn films fluctuates between 4.14 and 4.18 eV, and the value for the 1:3 sample is 4.17 eV (Figure 2f). It can effectively reduce the cathode WF and cascade with the lowest unoccupied molecular orbital (LUMO) of ITIC‐Th, which facilitates the transport and collection of electrons in OPDs (Figure S1, Supporting Information). The energy of the highest occupied molecular orbital (HOMO) of PEIE‐Sn, −7.62 eV versus vacuum (Text S1, Supporting Information),^[^ ^25^ ^]^ can effectively block the injection holes from external circuits.

Maintaining a high photocurrent density (*J_ph_*) while minimizing the dark current density (*J_d_*), which can span multiple orders of magnitude depending on the material properties and device architecture, is a prerequisite for obtaining OPDs with high detectivity. The *J_ph_* and *J_d_* of OPD, extracted from the current density‐voltage (*J–V*) characteristic curves (Figure S12a, Supporting Information) under different reverse biases, are used to study the capability of light response and dark current suppression (Figure 2h). As a result, the 1:3 PS‐OPD delivers the highest on/off ratio of more than 5 orders of magnitudes (*λ* of 650 nm, light intensity of 0.74 mW cm^−2^), and the *J_d_* is effectively suppressed due to the high injection barrier and the smooth interface. What is more, in order to eliminate the influence of thickness on the characteristics of CIL with different M_N_/M_Sn_, we prepared OPDs with different film thicknesses of CIL by multiple spin coating and their performance was compared (Figure S12b,c, Supporting Information). The results indicate that the PEIE‐Sn of 1:3 is the best, and thus further research is conducted. It is worth noting that when the PEIE content is high, the open circuit voltage of devices in the dark characteristic shifts as the film thickness increases (Figure S12b, Supporting Information), which can be explained by the displacement current connected with the polarization effect of the PEIE dipoles.^[^ ^42^ ^]^

Some physical phenomena, such as the diffusion of the electrode atoms into the active layer and the introduction of impurities during the device fabrication process, will seriously affect the device's stability.^[^ ^43^, ^44^ ^]^ We used time‐of‐flight secondary ion mass spectrometry (TOF–SIMS) to probe the elemental distribution and interface boundary in each layer of the PS‐OPD. The variation curve of the characteristic element with sputtering time shows abrupt delamination between layers (Figure 2i), typically for the PEIE‐Sn film (inset of Figure 2i), indicating that the underlying layer suffered no damage from the latter film preparation process.

### 2.2 Interface Characteristics of PS‐OPD as Compared to its Counterparts

**Figure 3:**
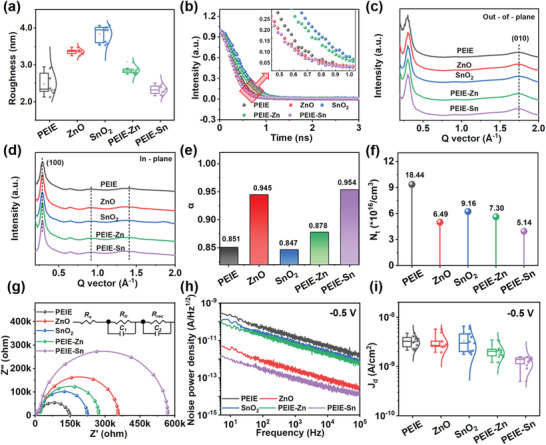
Comparison of charge carrier transmission and recombination dynamics in different CILs. a) Box plots of roughness. b) TRPL spectra of active layers on different CILs. The corresponding GIWAXS profiles along the c) out‐of‐plane and d) in‐plane directions. Comparison of the e) *α*‐value, f) the defect density (*N_t_*), g) the EIS Nyquist plot, and h) the noise spectral density of OPDs with various CILs. i) Box plots of *J_d_* of OPDs at −0.5 V.

The interface characteristic has a significant impact on the photoelectric characteristic of OPDs. Here, we compared five typical CILs, namely PEIE, ZnO, SnO_2_, PEIE‐Zn, and PEIE‐Sn proposed in this work, from the microscopic interface phenomena to the macroscopic device performance. We prepared different CIL films on ITO electrodes according to the process commonly used in the literature, and **Figure** **3**a exhibits the distribution statistics of ITO/CIL film surface roughness at diverse positions (extracted from the atomic force microscopy (AFM) topologies of Figure S13a, Supporting Information). It is worth noting that the roughness of the typical counterparts (ZnO, SnO_2_, and PEIE) thin films can match the level of literature.^[^ ^45^, ^46^ ^]^ The experimental results demonstrate that PEIE‐Sn can effectively polish the surface roughness of ITO, and the negligible variation in roughness at different regions indicates that the film is uniform, which benefits ameliorating the quality of the active layer and allows for better charge extraction. Additionally, PEIE‐Sn has a much larger contact angle as compared to its counterparts (Figure S13b, Supporting Information), which may be beneficial for improving the uniform, conformal, and close contact between PEIE‐Sn and the photoactive layer.

We deposited BHJ films onto the above‐mentioned CILs and investigated their interfacial charge extraction ability via the steady photoluminescence and time‐resolved photoluminescence (TRPL) decay characterization (with an excitation at 375 nm and emission at 774 nm). The steady‐state PL results suggested that PEIE‐Sn/BHJ had a much lower fluorescence intensity among all the samples (Figure S14, Supporting Information). The photoluminescence decay time and amplitudes were fitted and estimated using the monoexponential function (Figure 3b). The TRPL spectrum of the PEIE‐Sn sample exhibits the shortest decay time of 0.298 ns (Text S1 and Table S2, Supporting Information), implying that efficient electron extraction at the interface.^[^ ^26^ ^]^

2D Grazing‐incidence wide‐angle X‐ray scattering (GIWAXS) analysis was conducted to investigate the crystalline ordering and orientation of BHJ films on different CILs. All the obtained diffraction images (Figure S15a–e, Supporting Information) present a similar GIWAXS pattern, and the highly oriented face‐on structure is revealed by the (010) π–π stacking diffraction peaks (*q_z_* ≈ 1.75 Å^−1^) in the out‐of‐plane direction (Figure 3c) and (100) lamellar stacking peaks (*q_xy_* ≈ 0.29Å^−1^) in the in‐plane direction (Figure 3d).^[^ ^47^ ^]^ For quantitative analyses, the stacking distance, crystalline coherence length (*L_C_*, calculated by Scherrer equation), and paracrystalline disorder parameters (g_(010)_ and g_(h00)_) were calculated and summarized (Text S1 and Table S3, Supporting Information).^[^ ^48^ ^]^ The π–π stacking *L_C_* differences of CIL/BHJ films are relatively small, with PEIE‐Sn being slightly larger. Additionally, the g_(010)_/g_(h00)_ of PEIE‐Sn/BHJ is 16.016/10.993%, slightly smaller than its counterparts (Table S3, Supporting Information). The low paracrystalline disorder and structural defect states can lead to higher charge carrier mobility and suppressed electron trapping from defects.^[^ ^49^ ^]^

The *J–V* curves of different CIL‐based OPDs at diverse light intensities (Figure S16a–e, Supporting Information) were measured, and the power law (*J_SC_* ∝ *I^α^*) described the relationship between *J_SC_* and *I* (short‐circuit photocurrent density and light intensity) was extracted (Figure S16f, Supporting Information). The exponent *α* indicates the bimolecular recombination coefficient, and *α*∼1 implies a smaller recombination loss. The large slope of PS‐OPD indicates that bimolecular recombination during sweeping out the charge carriers is effectively suppressed at the CIL/BHJ interface (Figure 3e). The space charge–limited current measurement was used to evaluate the defect density (*N_t_*) on electron‐only devices with different CILs (Figure S17, Supporting Information). The calculated *N_t_* in PS‐OPD is 3.95 × 10^16^ cm^−3^, smaller than that of its counterparts (Figure 3f), suggesting that the target OPD has the lowest trap‐assisted recombination. We further investigated the interfacial charge transport behavior of the OPDs by using electrochemical impedance spectroscopy (EIS). The Nyquist plots measured in the dark and near open circuit voltage condition (0.5 V) are obtained, which also include the corresponding fitted curves based on a commonly used equivalent circuit model (Figure 3g). The parameters used for fitting are summarized in Table S4, Supporting Information, among which *R_tr_* (contact resistance) and *R_rec_* (recombination resistance) reflect the electron transport and electron–hole recombination, respectively.^[^ ^50^ ^]^ The PS‐OPD delivers the largest *R_rec_* value among all the devices, indicating that the electron‐hole recombination process is efficiently blocked at the PEIE‐Sn/BHJ interface.

The dark characteristics of different OPDs were investigated by analyzing the noise current and *J–V* curves. The PS‐OPD shows the lowest noise power density (Figure 3h) and slowly increased *J_d_* under applied reverse bias (Figure S18a, Supporting Information), indicating its superior interface characteristic and strong ability of *J_d_* suppression. The PS‐OPD exhibits a concentrated and smaller numerical distribution in *J_d_* statistics (Figure 3i and Figure S18b, Supporting Information), this superior batch consistency is speculated to be attributed to the smooth interface and low defect density. In the visible region, all the devices show a high external quantum efficiency (EQE) of over 60% (Figure S18c, Supporting Information), and the PS‐OPD shows the highest D* exceeds 10^13^ Jones (Figure S18d and Text S1, Supporting Information).^[^ ^51^ ^]^

### 2.3 Stability of PS‐OPD as Compared to its Counterparts

**Figure 4:**
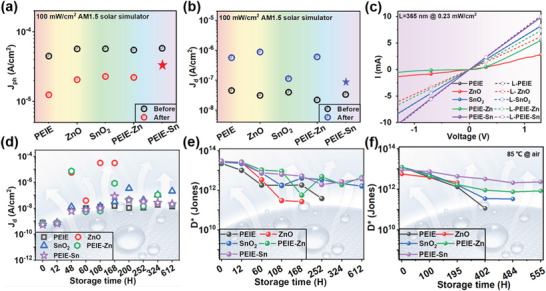
Comparison of stability of OPDs with different CILs. a) *J_ph_* and b) *J_d_* of OPDs with different CILs before and after solar simulator illumination (−2 V). c) Dark *J−V* characteristics before and after UV (365 nm) illumination of ITO/CIL/Ag devices. d) *J_d_* and e) D* evolution of unencapsulated devices based on different CILs stored in air conditions. f) D* tracking of unencapsulated OPDs with different CILs under 85 °C in air.

Next, we investigated the stability of different CIL‐based OPDs. The device photostability was studied by 1‐h continuously exposing the OPDs to a solar simulator (100 mWcm^−2^) and studying the variation in their *J_d_–V* and *J_ph_–V* characteristics (Figure S19, Supporting Information). As to the *J_ph_* (**Figure** **4**a), the attenuation of PS‐OPD is not remarkable as compared to its counterparts, which may be due to the aforementioned superior interfacial properties. Furthermore, the *J_d_* of SnO_2_ and PS‐OPDs is found to be close to pristine levels after illumination, in contrast to the PEIE, ZnO, and PEIE‐Zn counterparts which increased by an order of magnitude (Figure 4b). It is worth mentioning that there is a sharp debasement for the PEIE device, indicating the poor interface compatibility between PEIE and NFAs.

To explore the variation in contact characteristic of the CIL/cathode interface when exposed to the light, devices with the configuration of ITO/CIL/Ag were fabricated and illuminated to typical UV (365 nm), visible (vis, 650 nm), and near‐infrared (NIR, 940 nm) lights, respectively. All the dark *J−V* curves show excellent consistency before and after Vis and NIR illumination (Figure S20a,b, Supporting Information). When exposed to the UV light (Figure 4a), devices based on PEIE‐Sn, PEIE, and SnO_2_ show symmetric linear *J−V* behavior and no conspicuous change in resistance, evidencing good ohmic contact of these CIL/electrode interfaces. In contrast, ZnO and PEIE‐Zn devices show repeatable change from non‐ohmic contact to ohmic one with exposure and removal of UV light (Figure S20c, Supporting Information). It means that the illumination‐induced inconsistency issue cannot only be simply circumvented by accepting a worse performance. The absorption profile of the five CILs (Figure S20d, Supporting Information) indicates that ZnO and PEIE‐Zn have strong absorption of UV light from 300 to 400 nm. The change in CIL properties caused by high UV absorption may also bring about serious photostability problems in practical applications.

Then, we evaluated the device's stability under air conditions. The time‐variation curves of D* and EQE of unencapsulated OPDs were measured (Figure S21a–j, Supporting Information), and the values at 720 nm (−0.5 V) were extracted and displayed in Figure 4e and Figure S21k, Supporting Information, respectively. Regarding the *J_d_* (Figure 4d), PEIE‐Sn and PEIE devices show much lower values and a relatively gentle rising trend, while the ZnO and PEIE‐Zn OPDs exhibit obvious fluctuation due to their sensitivity to oxygen and moisture. Especially, the *J_d_* of ZnO‐OPD increases from 6.1 × 10^−10^ to 3.9 × 10^−5^ A cm^−2^ after exposure of 168 h, resulting in an on‐off current ratio close to 0 and loss of optical signal detection capability (Figure S21l, Supporting Information). The D* of PS‐OPD fluctuates slightly, maintaining 3.8 × 10^12^ Jones after 612 h of exposure to air, showing a relatively stable change compared to its counterparts (Figure 4e).

Apart from photostability and air stability, the thermal stability of devices with different CIL was studied by tracking the performance of the devices under thermal annealing at 85 °C in air. After 555 h, the attenuation of D* in PS‐OPD is relatively moderate and remains around 3 × 10^12^ Jones (Figure 4f). On the contrary, the performance of PEIE, ZnO, and SnO_2_ OPDs deteriorates severely, and they cannot effectively work due to the sudden rise of *J_d_*, which is consistent with the air stability mentioned above. It is worth mentioning that the environment temperature difference may cause a slight fluctuation in the initial D* of the device (Figure 4e,f), which can be ignored because the trend of device performance attenuation is more noteworthy. Considering the test results of photostability, air stability, and thermal stability, PEIE‐Sn stands out as the CIL of NF‐OPD due to its light insensitivity, excellent interfacial properties, and chemical compatibility.

The non‐covalent interaction of PEIE‐Sn with ITIC‐Th was investigated by DFT, with PEIE as a control. The fragment of ITIC‐Th with a single electron (ITIC‐Th′^−^) was selected as the representative NFA (Figure S22a, Supporting Information). The ‐NH and ‐OH groups in PEIE (Figure S22b, Supporting Information) were chosen as the interaction sites, which have been proven to chelate tin ions (Figure S22c, Supporting Information) to form stable N–Sn and O–Sn bonds.^[^ ^34^ ^]^ The interaction energy between PEIE‐Sn and ITIC‐Th optimized by the DFT method is about −9.98 kcal mol^−1^, which is higher than that of their counterpart (Figure S22d–i, Supporting Information). And the calculated distance between PEIE‐Sn and ITIC‐Th′^−^ is much smaller than that of PEIE and ITIC‐Th′^−^ (Figure S22f,i, Supporting Information), the strong interaction force between PEIE‐Sn and ITIC‐Th′^−^ might promote the formation of a high‐quality interface between the PEIE‐Sn and BHJ to some extent. To further understand the adsorption capacity of PEIE and PEIE‐Sn for water molecules, theoretical calculations were performed using the b3lyp/6‐31 g and b3lyp/def2‐svp pseudopotential basis sets based on DFT, respectively.^[^ ^52^ ^]^ Figure S23e, Supporting Information shows that the adsorption energy of PEIE‐Sn for water is −4.016 kcal mol^−1^, which is smaller than that of PEIE (−12.676 kcal mol^−1^, Figure s23d, Supporting Information), indicating that PEIE‐Sn can effectively enhance the air stability of OPD.

### 2.4 Response Performance of PS‐OPDs

**Figure 5:**
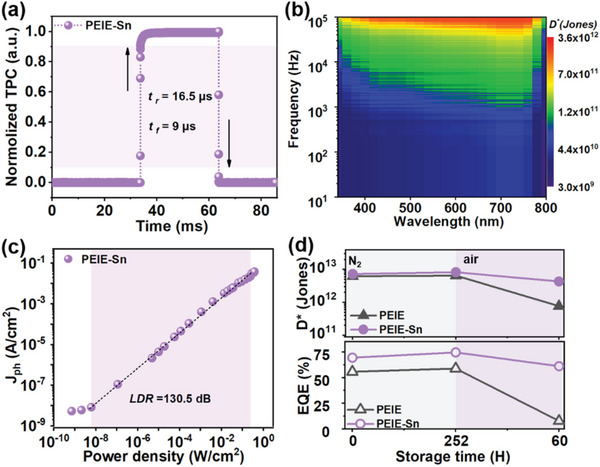
Characteristics of OPDs with PEIE‐Sn CIL. a) Transient photocurrent curve under 650 nm monochromatic light. b) D* as a function of frequency and incident light wavelength at −0.5 V. c) LDR under the illumination of 650 nm monochromatic light. d) Comparison of stability (D*, EQE) between PEIE‐OPD and PS‐OPD based on IT‐4F NFA.

Response time is also an important parameter in OPD, especially in the field of imaging. The response time of PS‐OPD was measured under the illumination of 650 nm (**Figure** **5**a), where the rise time (*t_r_*) and fall time (*t_f_*) are 16.5 and 9 µs (−0.5 V), respectively. The response time of other OPDs displays that their *t_f_* is longer than *t_r_* (Figure S24, Supporting Information), and the falling edges are significantly delayed, which may be related to the higher *N_t_* at the interface.^[^ ^53^, ^54^ ^]^ In addition, D* based on the noise current spectral density (*S_n_*) of the devices was also investigated in the test frequency range of 0.1 Hz to 100 kHz. The noise is dominated by 1/*f* in the low‐frequency range and shot noise gradually dominates in the high‐frequency range (Figure S25, Supporting Information). Due to the low *S_n_* (≈10^−14^ A Hz^−1/2^), the D* of PS‐OPD (Figure 5b) remains above 10^12^ Jones (340 to 800 nm, −0.5 V) in the relatively high‐frequency region, outperforming other counterparts (Figure S26, Supporting Information). The relationship between the *J_ph_* (−0.5 V) and incident optical power (650 nm) was plotted to study the linear dynamic range (LDR) of OPD (Text S1, Supporting Information).^[^ ^55^ ^]^ Owing to the low *J_d_*, PS‐OPD shows a high LDR of about 130.5 dB (Figure 5c), better than its counterparts (Figure S27, Supporting Information).

The universality of PEIE‐Sn was verified by applying it to other NF‐OPDs (IT‐4F and Y6), and the device stability was evaluated under N_2_ and air atmospheres. For the PEIE‐OPD, the device performance changes significantly after only 60 h of storage in air. As a comparison, the PS‐OPDs show a relatively stable performance (Figure 5d and Figure S28, Supporting Information).

## 3 Conclusion

In summary, we developed an organic metal chelating material (PEIE‐Sn) as the interface modification layer to improve the performance of NF‐OPDs. The Sn‐N/Sn‐O chelating effect in PEIE‐Sn can effectively inhibit interface chemical reactions between PEIE and NFAs, and contribute to the interface compatibility between functional layers. The PEIE‐Sn effectively promotes carrier transport and extraction by reducing the electrode WF and optimizing the energy level alignment. It also reduces the electrode surface roughness and interface defects, thereby suppressing carrier recombination. The above‐mentioned merits of PEIE‐Sn favorably contribute to improving the device's stability and photoelectric characteristics. As a result, the unencapsulated PS‐OPDs show superior detection capability and remain relatively stable performance after exposure to air, light, and heat. The design strategy of organometallic chelation proposed in this work has general utilities in various types of organic optoelectronic devices and may contribute valuable advances for increasing their commercialization prospects.

## 4 Experimental Section

### Materials

All the chemical reagents used in this work are of analytical grade without further purification. PBDB‐T, ITIC‐Th, IT‐4F, and Y6 were purchased from Solarmer Materials Inc. PEIE (80% ethoxylated solution, and 37 wt.% in H_2_O), zinc acetate dihydrate, chlorobenzene (CB), and SnO_2_ ink (2.5 wt.% in isopropyl alcohol) were purchased from Sigma‐Aldrich. Tin (II) acetate (Sn(Ac)_2_), N, N‐dimethylformamide (DMF), acetic acid (HAc), methoxyethanol monoethanolamine (MEA), MoO_3_, and Ag were purchased from Aladdin. ITO substrates (sheet resistance of 15 Ω sq^−1^) were purchased from South China Xiangcheng Technology Co., Ltd.

### Preparation of the CIL or Precursor Solutions

PEIE was dissolved in methoxyethanol to obtain a 0.3 wt.% solution, and SnO_2_ ink was dissolved in isopropyl alcohol to obtain a concentration of 0.5 wt.%. The ZnO precursor was prepared by dissolving zinc acetate dihydrate and MEA (M_N_/M_Sn_ of 1:1) in methoxyethanol, to form a sol‐gel solution. To prepare the PEIE‐Zn solution (M_N_/M_Zn_ of 1:9), zinc acetate dihydrate was added into the 0.3 wt.% PEIE solution. For PEIE‐Sn solutions, Sn(Ac)_2_ (15 mg ml^−1^, in *V_DMF_*:*V_Hac_* = 10:1) solution was blended with 0.3 wt.% PEIE solution with different ratios and stirred overnight.

### Fabrication of the Organic Photodiodes

The OPDs were fabricated with an inverted architecture of ITO/CILs/BHJ/MoO_3_/Ag. The ITO substrates were sonicated in deionized water, acetone, and isopropanol subsequently, and then dried with a nitrogen stream and treated with UV‐ozone for 20 min before use. The CIL layers were obtained by spin‐coating the CIL/precursor solutions (4000 rpm, 40 s) and then annealing (15 min at 120 °C for PEIE, PEIE‐Zn, and PEIE‐Sn, 30 min at 200 °C for ZnO and SnO_2_) under atmosphere. For the active layer blend solution, the PBDB‐T:NFAs (ITIC‐Th, Y6, or IT‐4F, weight ratio of 1:1) were dissolved in CB and stirred overnight at 50 °C under N_2_. Afterward, the substrates were transferred into the N_2_‐filled glovebox, and the active layer was spin‐coated (2000 rpm, 40 s) using the blend solution and then thermally annealed (120 °C, 10 min). Finally, the MoO_3_ layer (≈10 nm) and Ag layer (≈100 nm) were sequentially thermally evaporated under 10^−4^ Pa.

### Characterizations

The active layer thickness was determined by an Ambios XP‐300 surface profiler. The film morphology was characterized by SEM (ZEISS Gemini SEM 300, Germany), AFM (Bruker Dimension Icon, Germany), and TEM (FEI Tecnai G2 F20 S‐TWIN, USA). GIWAXS measurements were conducted by the Xeuss SAXS/WAXS system. The optical transmittance was measured with a UV–vis spectrometer (Varian, Cary5000). XPS measurements were conducted by the AXIS Supra XPS system. The steady‐state and TRPL spectra were obtained by Edinburgh FLS980 applied with an excitation wavelength of 375 nm. Tof‐SIMS measurement was performed using a TOF‐SIMS instrument (IONTOF GmbH, 169 Cameca IMS 4F). The WF was obtained from the onsets of the secondary cut‐off region (*E_cut‐off_*) by using UV photoelectron spectroscopy (Thermo Scientific, Multilab‐2000). AC impedance spectroscopy was carried out using an impedance analyzer compact state electrochemical interface (IVIUM Technologies). The current‐voltage characteristic was measured at RT (≈25 °C) with a Keithley 4200 semiconductor characterization system. The EQE and D* spectra were carried out with a Keithley 2636B source meter under a 150‐watt Xenon light source (Gloria‐X150A, Zolix Instruments) coupled with a monochromator, calibrated with a standard Si detector (S1337‐1010BQ, Hamamatsu Photonics). The noise spectral density and response time were measured by a semiconductor parameter analyzer (FS‐Pro, Primarius Technologies). For LDR measurement, a LED of 650 nm was used as a light source and a series of filters were used to modulate the incident light intensity.

## Conflict of Interest

The authors declare no conflict of interest.

## Supporting information

Supporting Information

## Data Availability

The data that support the findings of this study are available from the corresponding author upon reasonable request.
