# Supplementary material for: Stabilizing Non‐Fullerene Organic Photodiodes through Interface Engineering Enabled by a Tin Ion‐Chelated Polymer
Source: Adv Sci (Weinh). 2023 Aug 4;10(28):2302976. doi: 10.1002/advs.202302976 (PMC10558641; doi:10.1002/advs.202302976)
Supplement: Supplementary file 1 — Supporting Information [file ADVS-10-2302976-s001.pdf]

ADVANCED SCIENCE
Open Access

Supporting Information

for *Adv. Sci.*, DOI 10.1002/advs.202302976

Stabilizing Non-Fullerene Organic Photodiodes through Interface Engineering Enabled by a Tin Ion-Chelated Polymer

Jianhua Xiao, Yang Wang, Liu Yuan, Yin Long, Zhi Jiang, Qingxia Liu, Deen Gu, Weizhi Li, Huiling Tai* and Yadong Jiang*

Supporting Information

Stabilizing Non-fullerene Organic Photodiodes Through Interface Engineering Enabled by a Tin Ions-chelated Polymer

Jianhua Xiao, Yang Wang, Liu Yuan, Yin Long, Zhi Jiang, Qingxia Liu, Deen Gu, Weizhi Li, Huiling Tai*, and Yadong Jiang*

Computational details

The energy level calculations:

The energy of the highest occupied molecular orbital (HOMO) of PEIE-Sn is calculated by Equation S1

$$\mathrm{HOMO} = hv - (E_{\mathrm{cutoff}} - E_{\mathrm{onset}}) \qquad \text{(Equation S1)}$$

where hv is the energy of the incident photon from the He (I) source (21.22 eV), E_{cutoff}, 17.05 eV as displayed in Figure 2g (right), is secondary electron cutoff edge, and E_{onset}, 3.45eV as shown in Figure 2g (left), is the valence region onset.

An optical bandgap ($E_{g,opt}$) of 3.55 eV is estimated from the Tauc plot (Figure S1a).

Combining the HOMO of 7.62 eV, the LUMO of PEIE-Sn is estimated to be 4.07 eV.

The time-resolved photoluminescence (TRPL) curve fitting:

The TRPL decay characterization for the PBDB-T:ITIC-Th active layer deposited on various CILs were carried out to study the charge extraction. The PL decay time and amplitudes are fitted and estimated using the monoexponential function Equation S2

$$f(t) = A_1 exp^{-t/\tau_1} + C \qquad \text{(Equation S2)}$$

where τ_1 and A_1 are the decay times and the pre-exponential constants or decay amplitudes respectively; C is a constant for the baseline offset.

Paracrystalline disorder (g) calculations:

From 2D-(Grazing Incidence X-Ray Diffraction) GIXD data, the paracrystalline disorder parameter for the (010) peaks ($g_{(010)}$) can be calculated by using the single peak-width estimation method based on

$$g_{(010)} = \sqrt{\frac{\Delta q}{2\pi q_0}} \qquad \text{(Equation S3)}$$

where the Δq and $q0$ is the width and center position of the diffraction peak, respectively. And the paracrystalline disorder parameter for the (h00) peaks can be calculated from the slope (m) of δb - h^2 plot (Figure S15F), which is determined by

$$m = \frac{{g_{(\mathrm{h00})}}^2 \cdot \pi^2}{d} \quad \text{(Equation S4)}$$

where δb is the integral width of the diffraction peak, h is the order of diffraction and d is the domain spacing.

Defect density calculations:

The electron-only devices with the configurations of ITO/CILs (cathode interfacial layers) /PBDB-T:ITIC-Th/PFN-Br/Ag were prepared to calculate the defect density. In the space charge–limited current (SCLC) regime, the current is dominated by charge carriers injected from the contacts and the current-voltage characteristics become quadratic (I~V^2). Figure S17 shows the *J-V* curves of the fabricated devices on a double logarithmic scale, which comprises the Ohmic region, the trap-filling limit (TFL) region and the Child region. In the TFL region, the trap-state density (N_t) can be calculated by the following Equation S5

$$N_t = \frac{2\varepsilon\varepsilon_0 V_{TFL}}{qL^2} \quad \text{(Equation S5)}$$

where ε and ε_0 are the relative dielectric constant and vacuum permittivity, respectively. V_{TFL} is the onset voltage of TFL region, q is elementary charge. L represents PBDB-T:ITIC-Th thin film thickness, which was calculated to 300 nm. The N_t of OPDs with PEIE, ZnO, SnO_2, PEIE-Zn, PEIE-Sn as CIL is 9.35×10^{16}, 4.99×10^{16}, 6.23×10^{16}, 5.61×10^{16} and 3.95×10^{16} cm^{-3}, respectively

The specific detectivity (*D) calculations:**

The D^* of a photodetector is one of the most important figure-of-merits that determines the sensitivity of a photodetector to optical signals, calculated by Equation S6

$$D* = \frac{R\sqrt{AB}}{\sqrt{I_n}} = \frac{R\sqrt{A}}{S_n} \quad \text{(Equation S6)}$$

where R, A, B, I_n, and S_n are responsivity, active device area, bandwidth, noise current and noise current spectral density, respectively. When the total noise of the device is dominated by the shot noise, D^* can be obtained by Equation S7

$$D* = \frac{R\sqrt{A}}{\sqrt{2qi_d}} = \frac{R}{\sqrt{2qJ_d}} \quad \text{(Equation S7)}$$

where q, i_d, and J_d are the electron charge, dark current, and dark current density, respectively.

The linear dynamic range (LDR) calculations:

The linear weak-light response range is always characterized by the LDR, defined as an optical power margin within which the output photocurrent is linearly proportional to optical signal input:

$$LDR = 20\,log_{10}\frac{L_{upper}}{L_{lower}}\ (dB) \qquad \text{(Equation S8)}$$

where L_{upper} and L_{lower} are the upper and lower limits of the light intensity in a particular range.

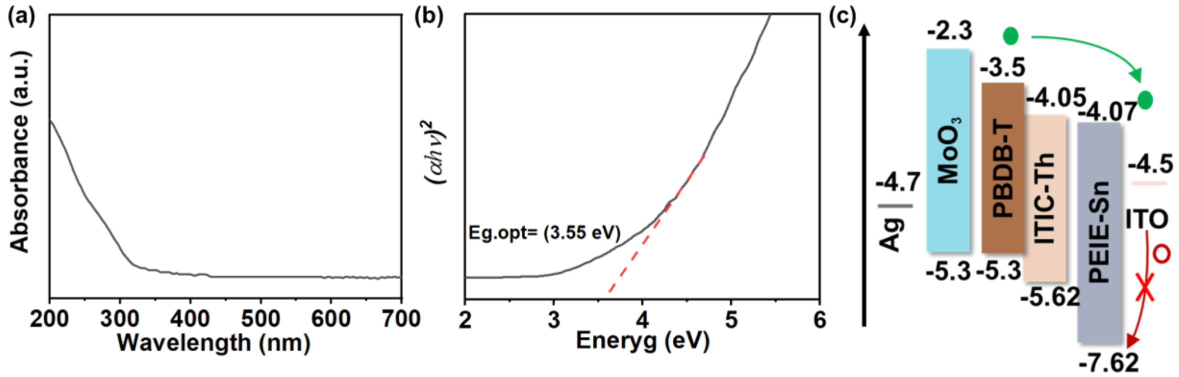

Figure S1. (a) Absorption spectrum of PEIE-Sn film on quartz. (b) $(\alpha hv)^2$ vs. photon energy. (c) The energy band diagram of our non-fullerene organic photodiode.

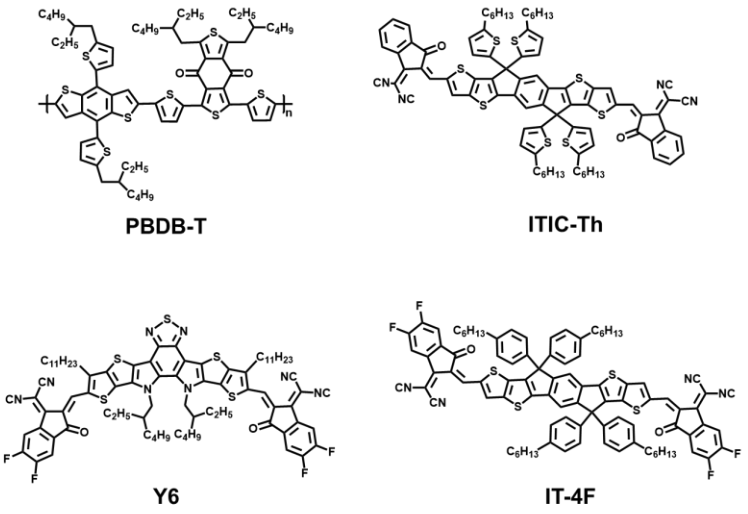

Figure S2. The chemical structural formulas of PBDB-T, ITIC-Th, Y6 and IT-4F.

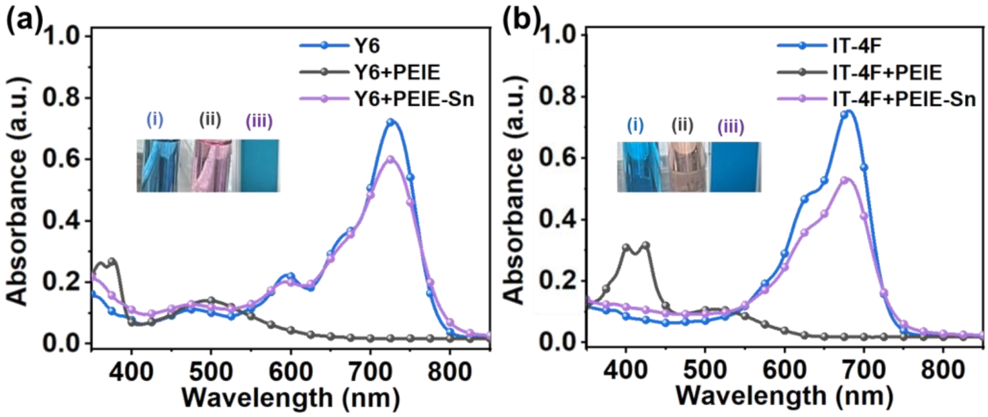

Figure S3. Absorption spectra and optical pictures (insets) of three solutions. (a) Y6, Y6: PEIE and Y6: PEIE-Sn, (b) IT-4F, IT-4F: PEIE and IT-4F: PEIE-Sn.

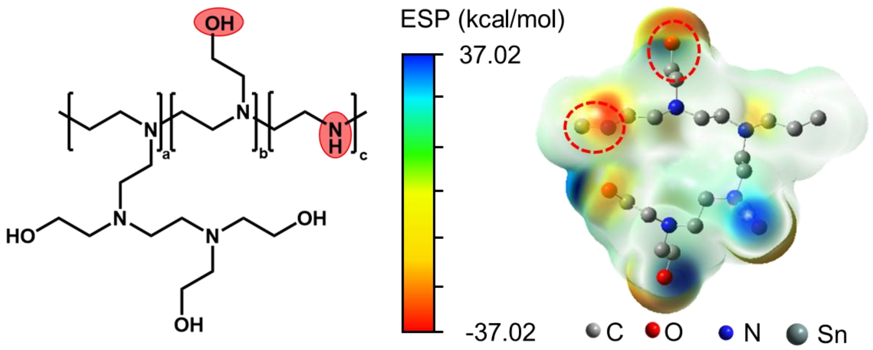

Figure S4. Electrostatic potential (ESP) analysis of PEIE. Chemical structure (left) and electrostatic potential distribution (right). The high electronegativity of O (-OH, -27.77 kcal mol^{-1}) and N (-NH, -28.64 kcal mol^{-1}) drive the formation of stable N-Sn and O-Sn bonds and significantly inhibit the chemical reaction between the PEIE and NFAs.

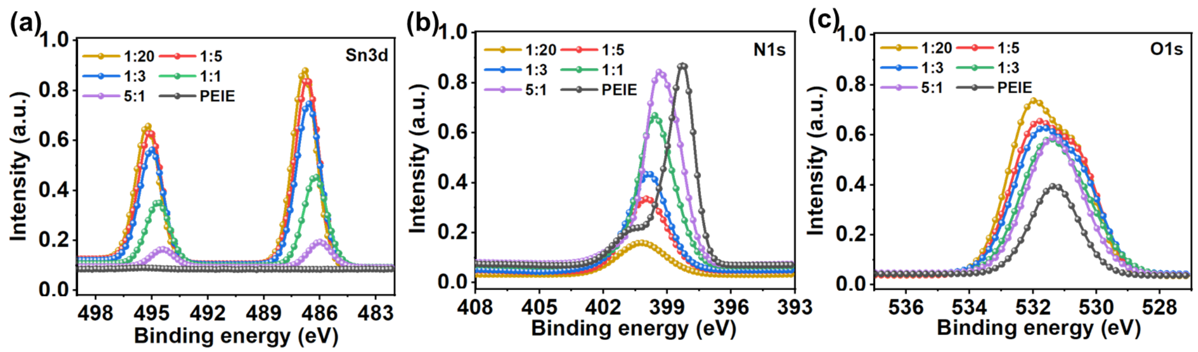

Figure S5. X-ray photoelectron spectroscopy of PEIE-Sn with different M_N/M_{Sn}. (a) Sn 3d, (b) N 1s and (c) O 1s.

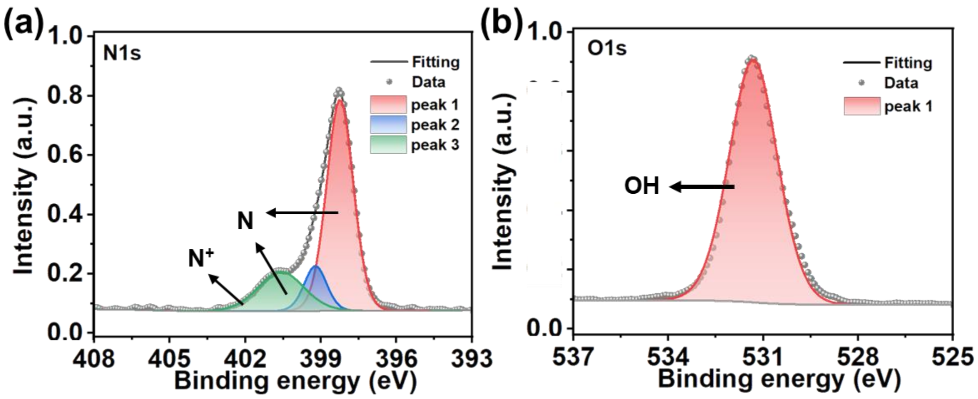

Figure S6. X-ray photoelectron spectroscopy of PEIE. (a) N 1s and (b) O 1s.

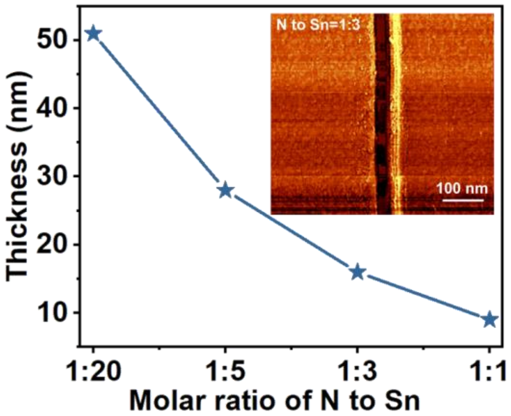

Figure S7. AFM diagram of PEIE-Sn films with different M_N/M_{Sn}.

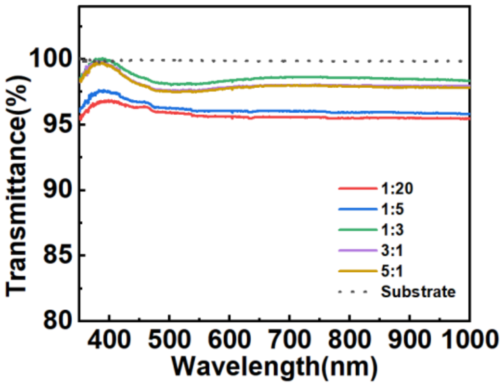

Figure S8. Transmittance spectra of PEIE-Sn films with different M_N/M_{Sn}.

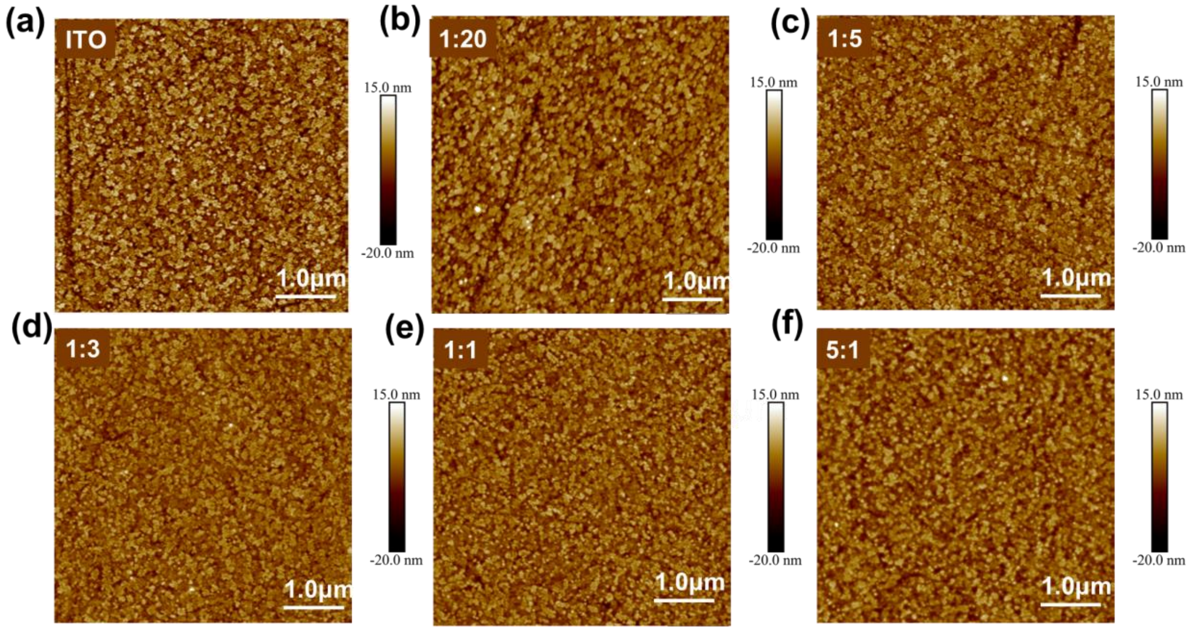

Figure S9. AFM topology profiles. (a) ITO. PEIE-Sn with different M_N/M_{Sn}: (b) 1:20, (c) 1:5, (d) 1:3, (e) 1:1 and (f) 5:1, respectively.

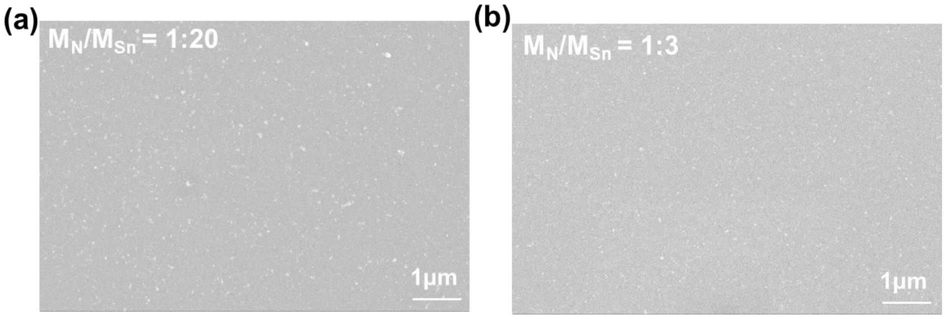

Figure S10. SEM images of PEIE-Sn with different M_N/M_{Sn}. (a) 1:20 and (b) 1:3.

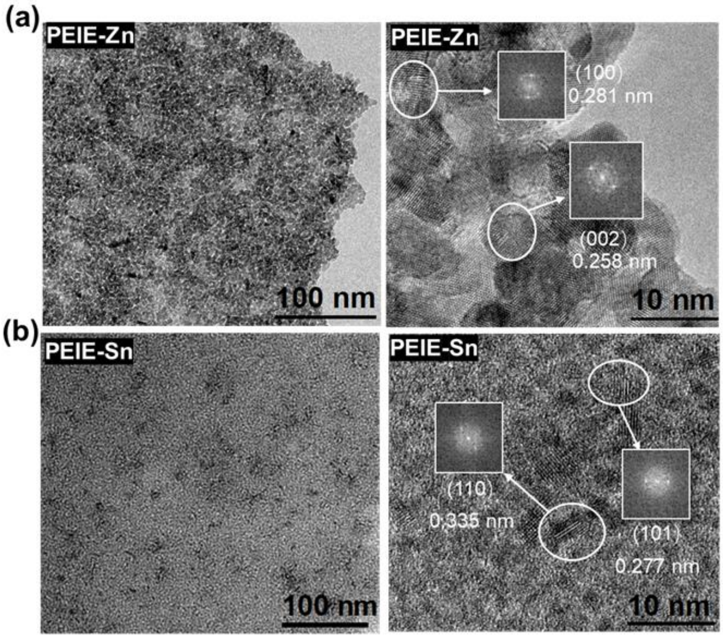

Figure S11. HRTEM images of (a) PEIE-Zn and (b)PEIE-Sn with M_N/M_{Sn} of 1:3.

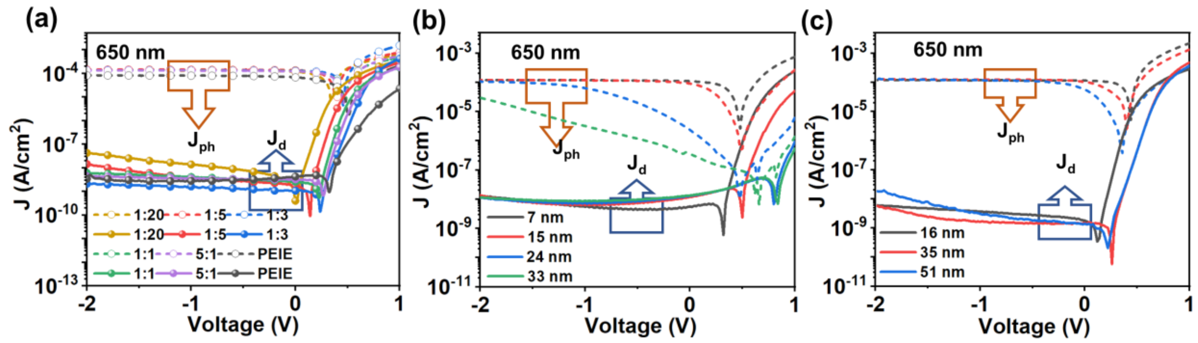

Figure S12. *J*-*V* characteristics of OPDs. (a) PEIE-Sn as the CIL with different M_N/M_{Sn}. PEIE-Sn with different thicknesses of (b) 5:1 and (c) 1:3.

Figure S12b shows that as the film thickness increases, the device response with PEIE-Sn of 5:1 significantly decreases, which is consistent with the previously discussed sensitivity of PEIE to average thickness. In addition, the device with thick PEIE-Sn (51 nm) of 1:3 shows superior performance.

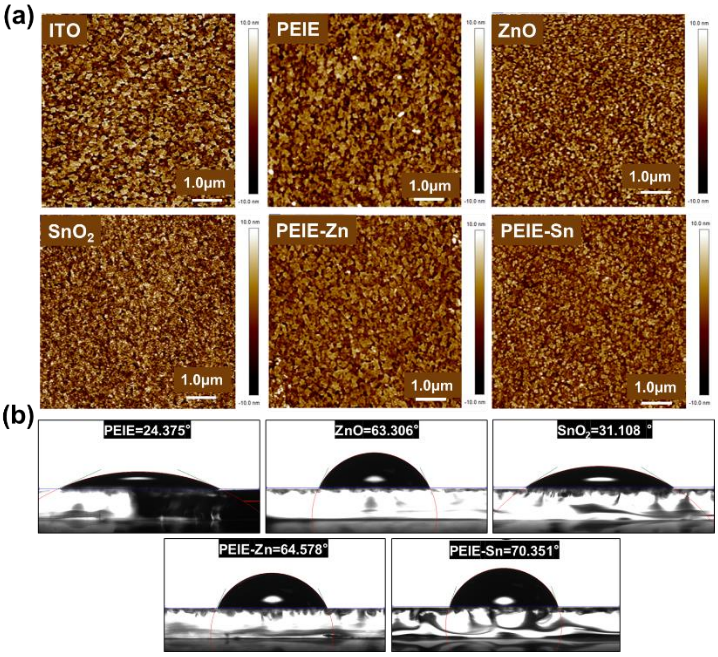

Figure S13. (a) AFM topology profile and (b) contact angles of different CILs with deionized water of ITO, PEIE, ZnO, SnO_2, PEIE-Zn and PEIE-Sn (M_N/M_{Sn} of 1:3), respectively.

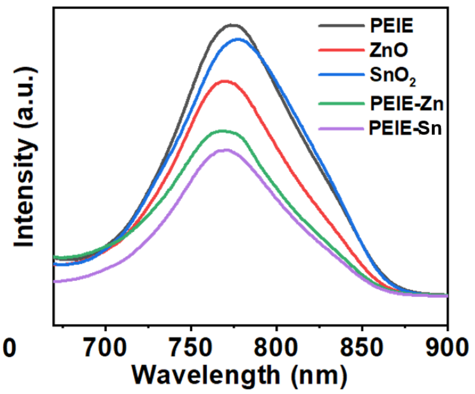

Figure S14. Photoluminescence spectra (PL) of the CIL/PBDB-T:ITIC-Th films.

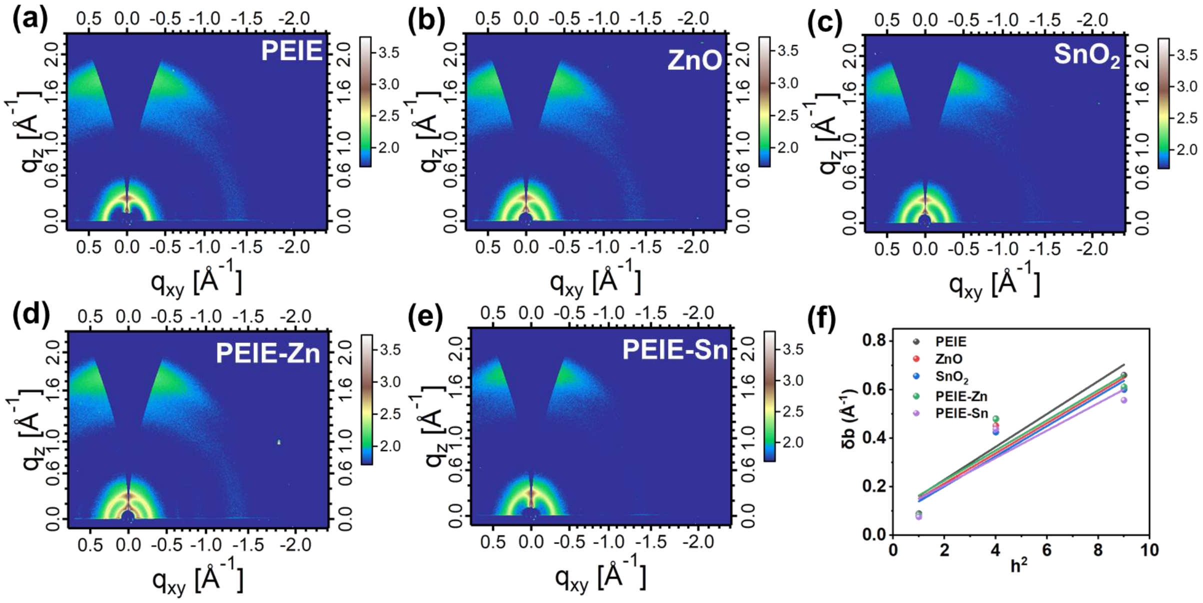

Figure S15. 2D-GIXD pattern images for different CIL/BHJ films. (a) PEIE, (b) ZnO, (c) SnO_2, (d) PEIE-Zn and (e) PEIE-Sn. (f) Fitting diagram of δb - h^2.

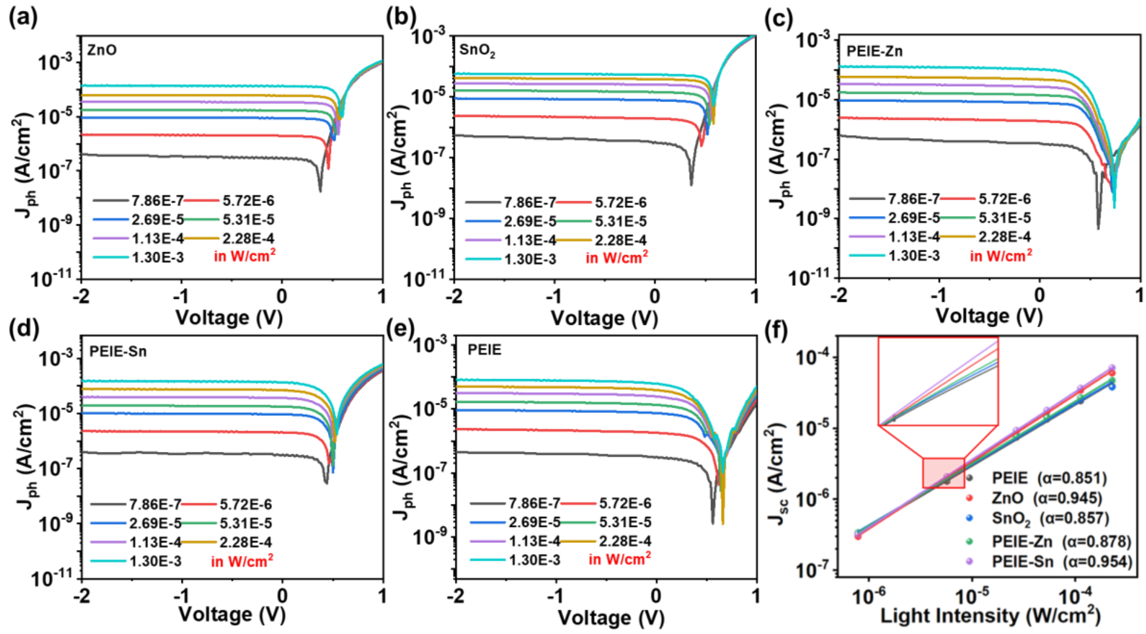

Figure S16. *J*-*V* curves of the OPDs with respect to light intensity. (a) PEIE, (b) ZnO, (c) SnO_2, (d) PEIE-Zn, (e) PEIE-Sn and (f) Light intensity dependent a J_{SC} of OPDs.

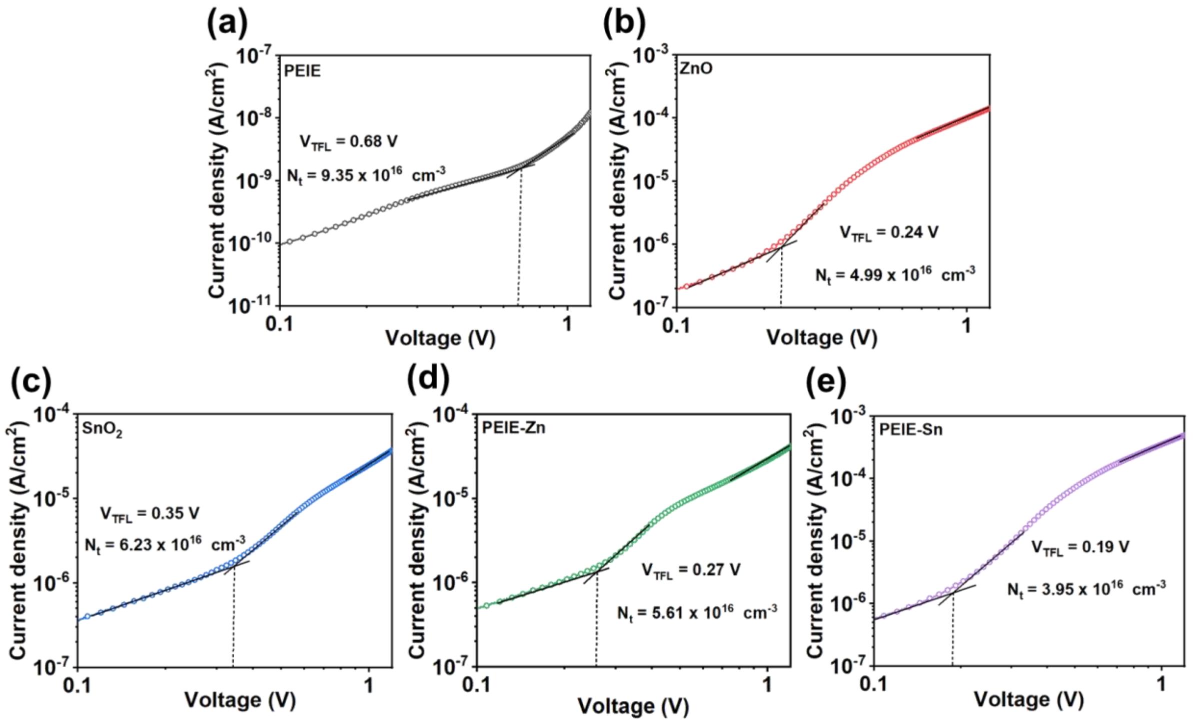

Figure S17. The space charge–limited current plots based on electron-only devices (ITO/CILs/PBDB-T: ITIC-Th/PFN-Br/Ag). (a) PEIE, (b) ZnO, (c) SnO_2, (d) PEIE-Zn and (e) PEIE-Sn.

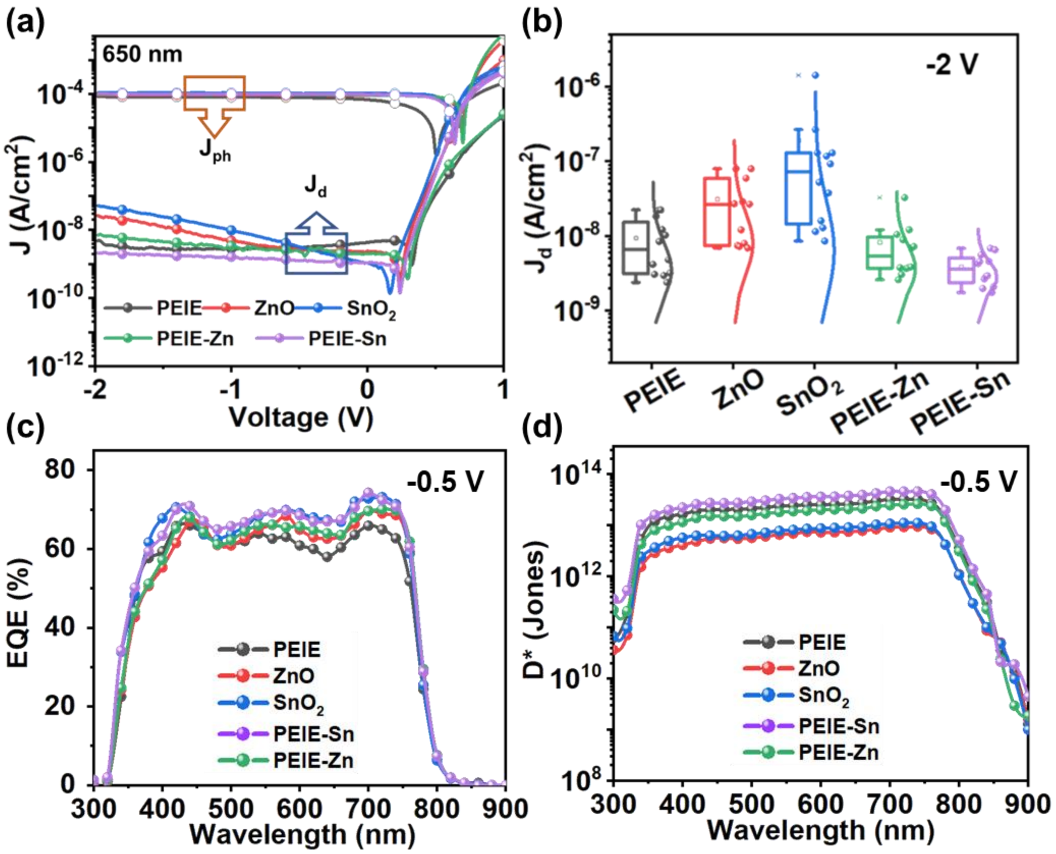

Figure S18. Performance of OPDs with different CILs. (a) *J*-*V* curves. (b) J_d at -2 V. (c) EQE and (d) *D** at -0.5 V.

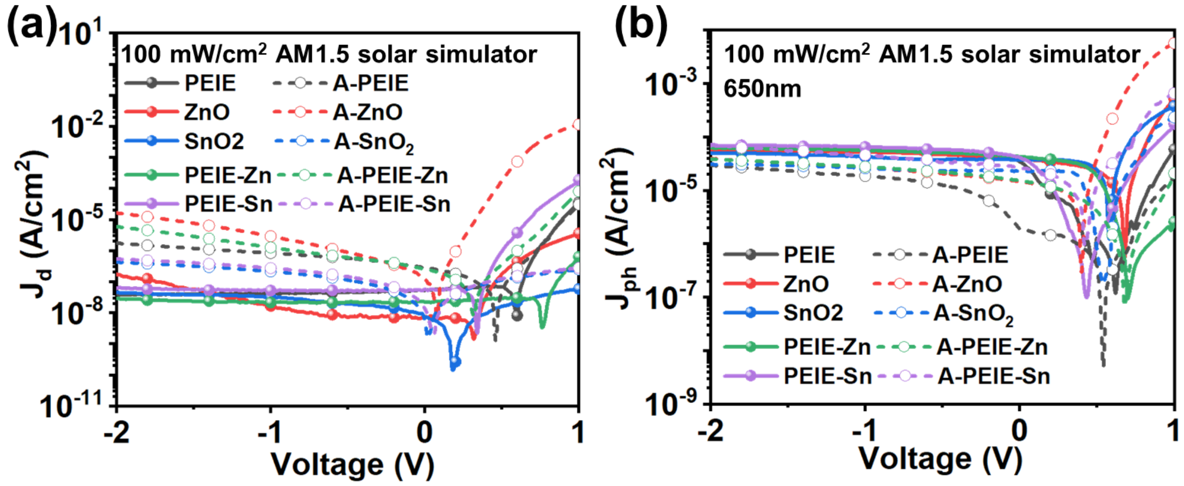

Figure S19. *J*–*V* characteristics before (solid lines) and after (short dashes) continuous illumination (100 mW cm^{-2}, AM1.5) under atmospheric condition for an hour. (a) J_{ph}–V and (b) J_d–V.

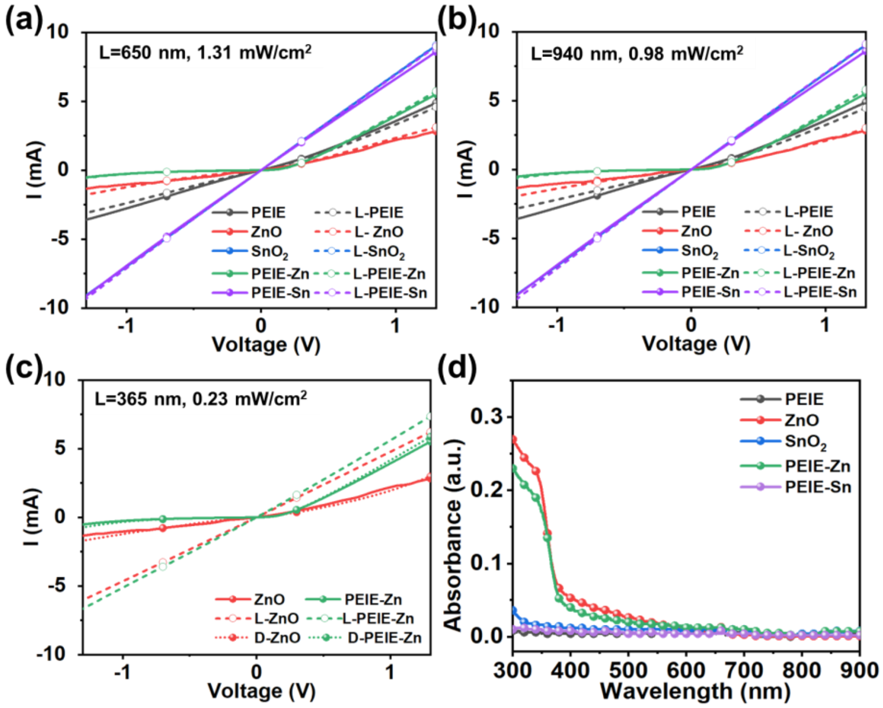

Figure S20. Dark $J-V$ characteristics before and after illumination of ITO/CIL/Ag devices. (a) Visible light (650 nm). (b) Near-infrared (940 nm). (c) Dark $J-V$ characteristics before (solid lines), after (short dashes) illumination, and leaving the ITO/ZnO or PEIE-Zn/Ag devices alone for a few hours (dotted lines). (d) Absorption spectra of CIL films on quartz.

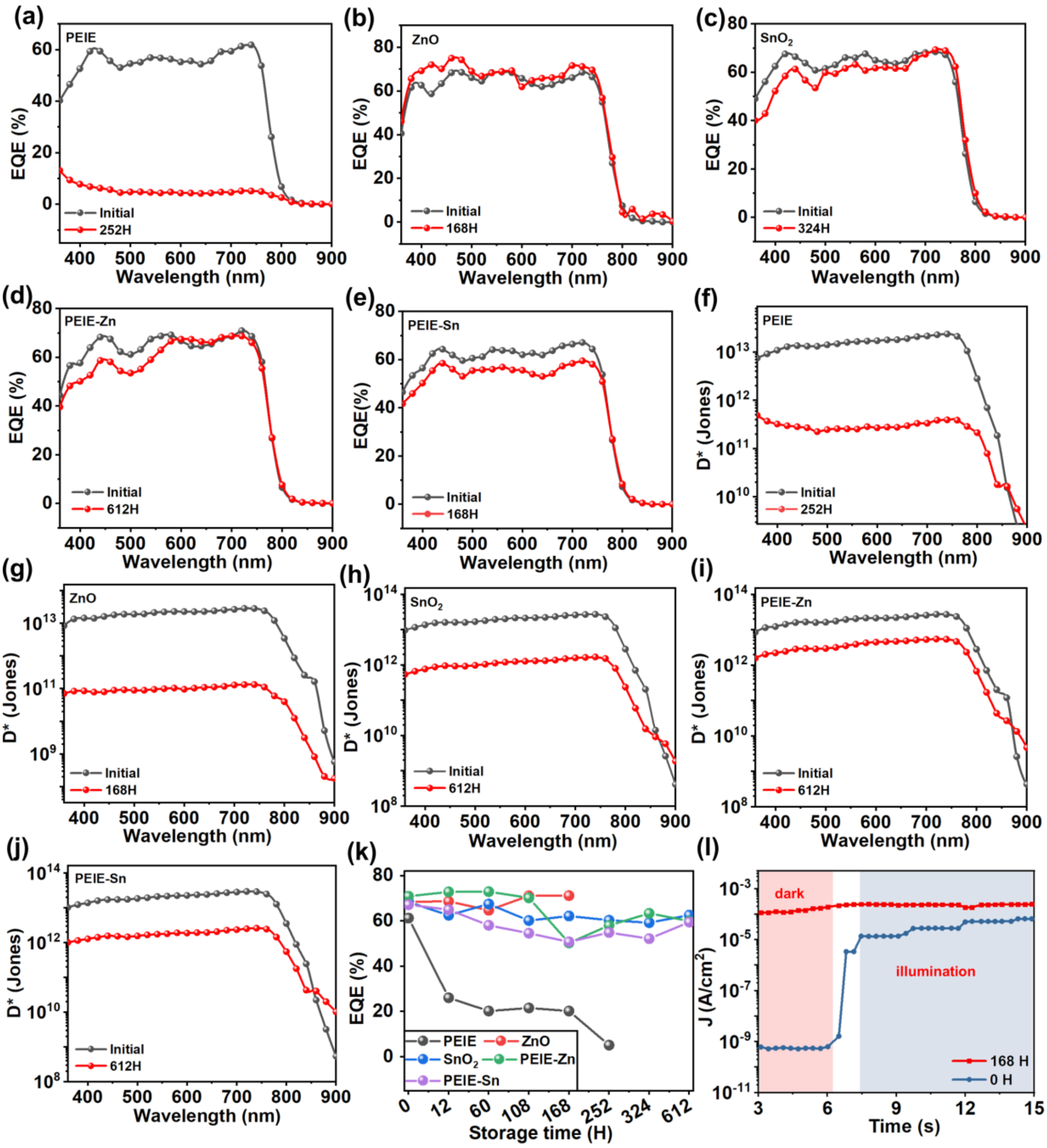

Figure S21. EQE-λ, D^*-λ and J-t at -0.5 V of unencapsulated devices. (a-e) EQE-λ and (f-j) D*-λ of PEIE, ZnO, SnO_2, PEIE-Zn and PEIE-Sn based NF-OPDs stored in atmospheric conditions, respectively. (k) EQE evolution of unencapsulated devices based on different CILs. (l) The current-time curves of ZnO based OPD at different storage time.

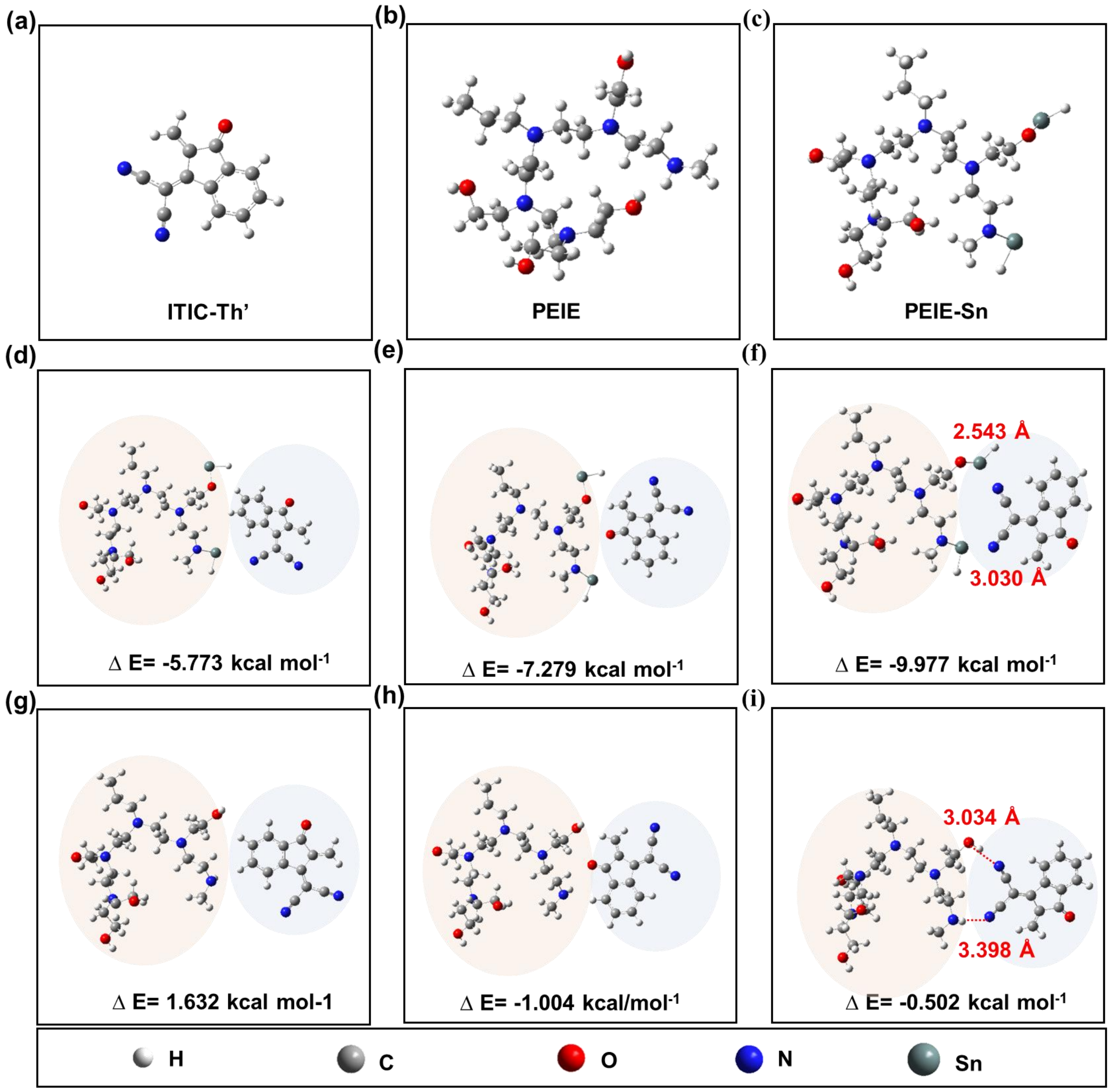

Figure S22. Different positions of ITIC-Th'- were selected as the interaction points with PEIE-Sn or PEIE, and the structures of various systems were optimized by the DFT method and their binding energies were calculated. Molecular structures of (a) ITIC-Th'-, (b) PEIE, (c) PEIE-Sn. The DFT calculation of (d-f) PEIE and (g-i) PEIE-Sn with ITIC-Th'-.

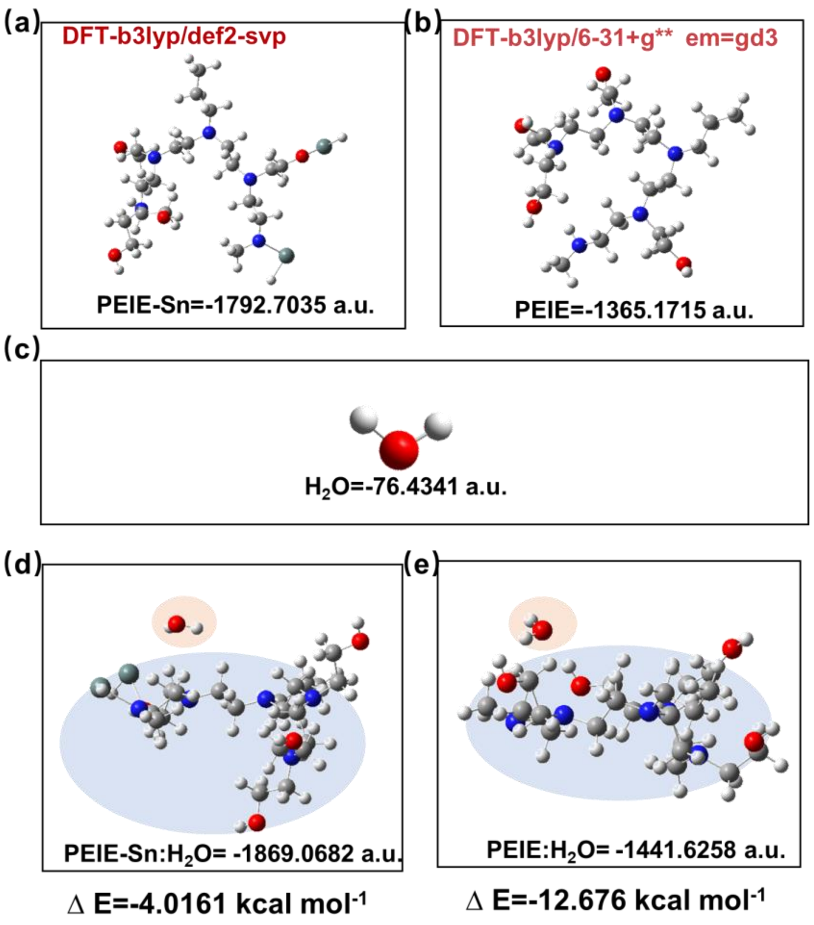

Figure S23. DFT calculation of the adsorption energy. (a) PEIE-Sn, (b) PEIE (c) H_2O, (d) PEIE-Sn-H_2O and (e) PEIE-H_2O.

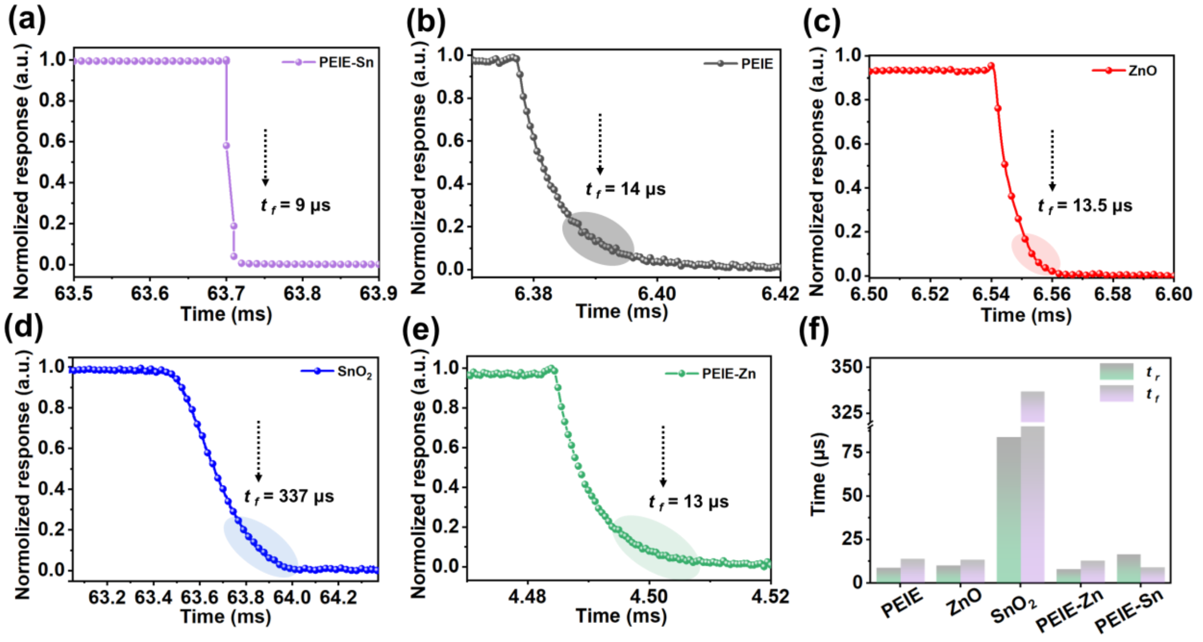

Figure S24. Response time statistics of devices based on different CIL. (a) PEIE-Sn, (b) PEIE, (c) ZnO, (d) SnO_2, (e) PEIE-Zn and (f) the comparison histogram.

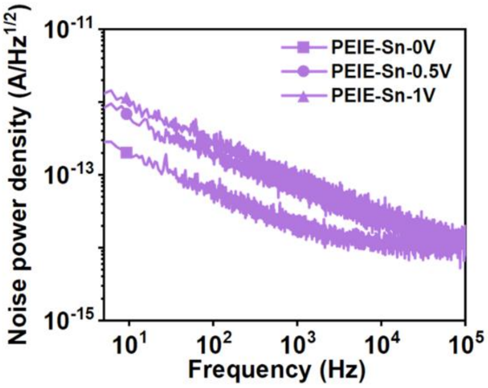

Figure S25. Noise spectral density of PS-OPD at different biases.

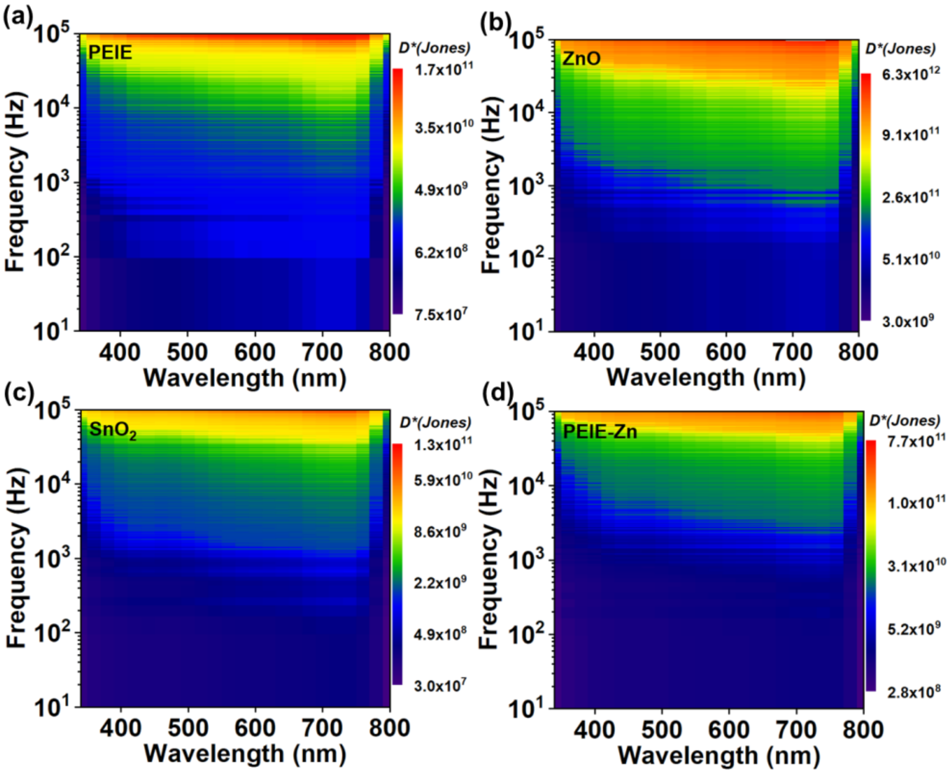

Figure S26. *D** as a function of frequency and incident light wavelength at -0.5 V for different CIL-based OPDs. (a) PEIE, (b) ZnO, (c) SnO_2 and (d) PEIE-Zn.

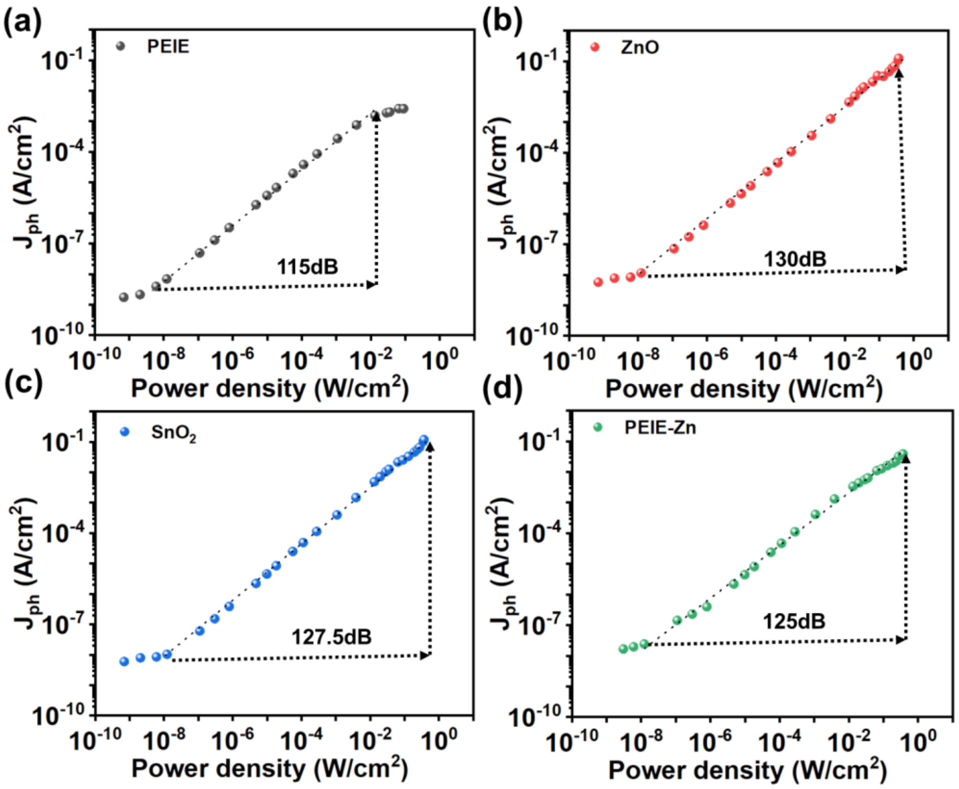

Figure S27. Linear dynamic range of OPDs with different CILs under the illumination of 650 nm monochromatic light. (a) PEIE, (b) ZnO, (c) SnO_2 and (d) PEIE-Zn.

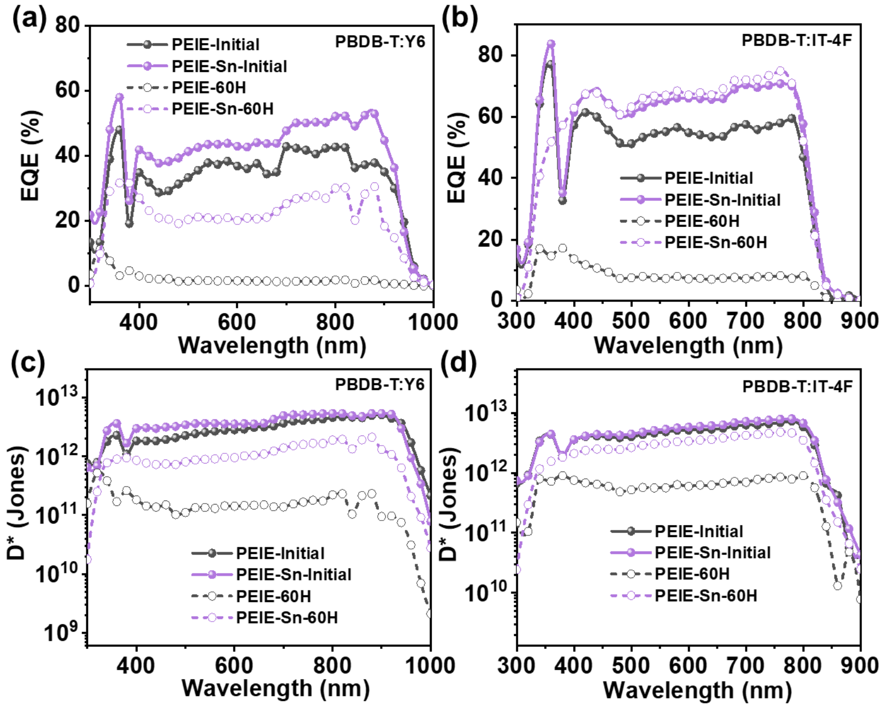

Figure S28. Stability of EQE and *D** (-0.5 V) of OPDs based on different NFAs stored in atmospheric conditions. (a, b) IT-4F. (c, d) Y6.

Table S1. Organic element analysis test report of PEIE.

Sample	N (%)	C (%)	H (%)	S (%)	O (%)
PEIE	11.125	34.256	10.79	0.154	41.358

Table S2. The fitted parameters of time-resolved photoluminescence (TRPL) spectra (excitation at 405 nm and emission at 774 nm) of PBDB-T:ITIC-Th layer deposited on different CILs.

ETL	τ_1(ns)	A1	X^2
PEIE	0.354	1.214	0.967
ZnO	0.303	1.165	0.982
SnO_2	0.472	1.196	0.975
PEIE-Zn	0.419	1.153	0.985
PEIE-Sn	0.298	1.167	0.983

Table S3. Summary of 2D-GIXD parameters of different CIL/BHJ films.

	In-plane (lamellar stacking)			Out-of-plane (π-π stacking)		
	d (nm)	L_c (nm)	$g_{(h00)}$ (%)	d (nm)	Lc (nm)	$g_{(010)}$ (%)
PEIE	2.162	6.958	12.166	0.359	2.140	16.330
	0.679	3.745				
	0.458	1.225				
ZnO	2.118	7.727	11.539	0.359	2.205	16.087
	0.693	4.035				
	0.458	1.190				
SnO_2	2.207	7.965	11.783	0.359	2.075	16.583
	0.670	3.572				
	0.458	1.141				
PEIE-Zn	2.207	7.430	11.765	0.368	2.196	16.324
	0.693	3.688				
	0.458	1.104				
PEIE-Sn	2.118	8.238	10.993	0.359	2.225	16.016
	0.693	3.527				
	0.458	1.239				

Table S4 Parameters extracted from fitting of the Nyquist plot of OPDs with various CILs.

ETL	$R_S(\Omega)$	$R_{tr}(\Omega)$	$C_1(F)$	$R_{rce}(\Omega)$	$C_2(F)$
PEIE	365.8	7903	1.46E-9	130280	3.44E-9
ZnO	706.5	26000	4.19E-10	329000	1.30E-9
SnO_2	480	18794	4.52E-10	204310	1.62E-9
PEIE-Zn	528.7	23113	7.71E-10	249230	6.53E-9
PEIE-Sn	743.6	19467	4.02E-10	548760	1.26E-9
